# Compression of high-density 0.16 pC electron bunches through high field gradients for ultrafast single shot electron diffraction: The Compact RF Gun

**DOI:** 10.1063/1.4979970

**Published:** 2017-04-12

**Authors:** Hazem Daoud, Klaus Floettmann, R. J. Dwayne Miller

**Affiliations:** 1 Departments of and Chemistry and Physics, University of Toronto, 80 St. George Street, Toronto, Ontario M5S 3H6, Canada; 2 DESY, Notkestrasse 85, 22603 Hamburg, Germany; 3 Max Planck Institute for the Structure and Dynamics of Matter, Luruper Chaussee 149, 22761 Hamburg, Germany

## Abstract

We present an RF gun design for single shot ultrafast electron diffraction experiments that can produce sub-100 fs high-charge electron bunches in the 130 keV energy range. Our simulations show that our proposed half-cell RF cavity is capable of producing 137 keV, 27 fs rms (60 fs FWHM), 10^6^ electron bunches with an rms spot size of 276 *μ*m and a transverse coherence length of 2.0 nm. The required operation power is 9.2 kW, significantly lower than conventional rf cavity designs and a key design feature. This electron source further relies on high electric field gradients at the cathode to simultaneously accelerate and compress the electron bunch to open up new space-time resolution domains for atomically resolved dynamics.

## I. INTRODUCTION

Directly observing atomic motions during structural transitions is one of the great challenges in science. In chemistry, this prospect represents a direct observation of passage through the transition state region, which is one of the unifying concepts.^1^ The particular nuclear configurations at this special moment in space-time define the barrier region, which is the target of control to direct chemistry along preferred pathways faster than competing processes.^2^ Experimental methods have been developed in recent years to enable such observations at the relevant spatial and temporal scales. The first use of structural probes to capture the structure of reactive intermediates and the importance of time resolved structural dynamics were the concepts of the work of Ischenko *et al.*^3^ This work used an elegant means to register microsecond electron pulses and 100% excitation levels to simplify diffraction analysis of photoinduced radicals. The use of photoinjection to obtain picosecond pulses was subsequently introduced.^4^ This approach was adopted by the Zewail group to carry out pioneering work on a number of classic gas phase reaction processes in which differential detection of diffraction patterns was introduced to capture reactive intermediates on few picosecond timescales.^5–8^ Given the very small signal to noise and low sample density of gas phase experiments, these experiments would still be considered major achievements. Independent work by Weber and Dudek introduced the use of high repetition laser sampling to improve the sensitivity and extend the studies to more complex systems.^9^ The time resolution in these early studies was sufficient for resolving the structure of intermediates but not for observing the specific atomic motion direction of the chemistry and passage through the barrier crossing region. These early experiments used beam geometries that required low electron bunch charge and were subject to velocity mismatch time broadening issues.^7^ The realization of high brightness electron sources to real time capture of atomic motions came from an effectively exact solution to the electron pulse propagation at sufficient bunch charge to enable the single shot structure determination of simple unit cell systems.^10,11^ This work effectively showed that despite coulomb repulsion or space charge issues, it was possible to design electron guns with sufficient spatial coherence, short pulse durations, and brightness (with respect to casting the challenge as an imaging problem) to resolve atomic motions in real time. Two electron source concepts came from this work, the implementation of compact electron gun designs and temporal refocusing of high bunch charge electron pulses. The introduction of high brightness electron sources has opened up the shooting of “molecular movies,” a sequence of atomic configurations at the relevant timescale during a specific chemical reaction. This new atomic window on chemistry has enabled a direct observation of the enormous reduction in dimensionality that occurs in barrier crossing to a few key modes^12^ and promises to provide a new conceptual basis for chemistry with respect to controlling barrier heights.^13^

Applications of these new experimental methods based on structural probes extend beyond the field of chemistry to the fields of biology and condensed matter physics.^2,14^ Two main methods are used for time resolving molecular dynamics: ultrafast x-ray diffraction^14^ and ultrafast electron diffraction (UED).^2,12,15^

X-rays, which are a form of electromagnetic radiation, interact with electrons, and thus, x-ray diffraction is a probe of the electron density of the sample. Electrons, on the other hand, are scattered by the electric potential of the sample which is formed by both the electrons and the nuclei. In comparison to x-ray diffraction experiments, ultrafast electron diffraction experiments are table top, the energy deposited per elastic scattering event is approximately 1000 times lower compared to 1.5 Å x-rays, and also for most samples, the scattering length of electrons better matches the optical penetration depth of the pump laser.^16^ Usually, 10^5^–10^6^ electrons per pulse are required for reasonably strong signals in electron diffraction experiments.

Despite that, femtosecond electron diffraction remains a challenging task as the Coulomb repulsive forces between the electrons cause electron pulses to expand both spatially and temporally, the aforementioned space charge effect. In recent years, however, several techniques have been developed to overcome this challenge. A traditional way to reduce the effect of the Coulomb forces is to accelerate electron bunches to relativistic energies of several MeVs using radiofrequency accelerators.^10^ However, this technique poses its own challenges in regard to the high financial expenses and broad technical expertise required to build and operate such a facility.

Because of these reasons and other considerations, the 100–300 keV energy range is a preferred region of energies for electron diffraction experiments. This approach avails a table top system that is fully capable of undergoing atomic resolved dynamics. One technique to counteract space charge effects is to reduce the number of electrons to one electron while increasing the repetition rate to several megahertz. This technique, however, requires the sample to be reproducibly pumped and probed ∼10^6^ times to obtain diffraction patterns of sufficient quality. The time resolution is mainly limited by the jitter in the arrival time of the electrons. This approach is similar in concept to femtosecond pump-probe experiments with 1-photon probe pulses. At a minimum, without signal to noise considerations for long integration times, this approach requires >10^6^ longer data acquisition times, which makes this problem intractable for most problems. There are examples in which the much larger spatial coherence of single electron sources has unique advantages for real space imaging, e.g., collective solid state effects.^17,18^ Generally, the brighter the electron source for a given application the better.

Another approach is to minimize the distance between electron emission and the sample, the compact gun design,^11^ so that the electron bunches have no time to expand. A third method is to accelerate a relatively long bunch, so that space charge effects in the cathode area are reduced and compress it longitudinally at higher energy.

For electron pulses in the 100–300 keV energy range, current acceleration technologies for single-shot ultrafast electron diffraction experiments make use of a DC voltage applied to a diode gap to reach the required energy followed by an RF cavity for compressing the electron bunch in the longitudinal direction to obtain electron bunches with time resolutions below 100 fs as required for resolving molecular dynamics.^19,20^ The average bond length is on the order of 1 Å, and since atoms typically move approximately at the speed of sound for the linear response to chemical forces (∼1000 m/s), 100 fs is the relevant timescale on which molecular dynamics take place.^21^ The additional requirement is related to the transverse coherence (vide infra) and depends on the complexity of the molecular system of interest, and typically 1 nm spatial coherence is sufficient.

Temporal compression requires a time varying RF field. Thus, this technique comes with its own challenges too. For optimal compression, electrons need to be injected into the RF field at a particular phase. Due to timing jitter, deviations from that phase result in uncertainty in the arrival time of the electrons at the sample, a factor that limits the overall time resolution of the obtained diffraction signals. The other major challenge is posed by the space charge effects between the electrons, which naturally put an upper limit on the number of electrons in the bunch. Typically, higher numbers of electrons per bunch are experimentally desired as they are directly proportional to the strength of the obtained signal. Current electron diffraction experiments face limitations in time resolution mainly due to the repulsive Coulomb forces between electrons. Thus, new concept designs enabling lower time resolutions are desired as they ‘unlock’ the yet undiscovered higher resolution domains of molecular dynamics and hence refine our understanding of the dynamic world we live in.

The purpose of this paper is threefold. (1) To present a novel RF gun design that requires only a relatively low power solid-state amplifier as the power source for the compression of high-density electron bunches. (2) To show through state-of-the-art computer simulations that the utilization of high electric field gradients is a promising method for the compression of high density electron bunches. (3) To show through our simulations that producing sub-100 fs pulses is possible under conditions and assumptions which are all within the scope of available technologies. The unique features of the present work are the extremely high extraction field possible with rf cavities, which directly translates to shorter electron pulses, and a novel cavity design to reduce the power requirements that would otherwise border on that used for relativistic electron pulse generation.

In Section II, we discuss the important parameters in regard to beam dynamics in single shot ultrafast electron diffraction experiments to provide the motivation for this development.

## II. SINGLE SHOT UED BEAM DYNAMICS

### A. General considerations

#### 1. Pulse duration

The pulse duration is an important parameter in UED experiments since it sets the limit for the maximum obtainable time resolution. During the pulse duration, electrons hit the sample and a diffraction pattern is formed over this time period. The shorter the pulse duration, the higher is the time resolution.

The time duration of the pulse is determined by two factors: the longitudinal length of the bunch and the electron velocity.

#### 2. Coherence length

The coherence length is defined as the maximum length between two electrons over which interference is still visible.^19^ The transverse coherence length is given by $L_{x}=\frac{\hbar }{mc}\frac{\sigma_{x}}{\varepsilon_{n,x}}$, where ℏ is Planck's constant divided by 2π, m is the mass of the electron, c is the speed of light, $\sigma_{x}$ is the rms beam size, and $\varepsilon_{n,x}$ is the transverse normalized emittance which is defined by $\varepsilon_{n,x}=\frac{1}{mc}\sqrt{\langle x^{2}\rangle \langle p_{x}^{2}\rangle -\langle xp_{x}\rangle^{2}}$, where the angular brackets ⟨⟩ indicate the average over the electrons in the bunch, and $\langle x\rangle =\langle p_{x}\rangle =0$ is assumed.

#### 3. Bunch charge

Based on practical experience, experimentally, a bunch containing ≥10^5^ electrons is needed for recording a diffraction signal with suitable contrast. This corresponds to a bunch charge of only ≥0.016 pC. More electrons give a stronger signal; however, the greater the bunch charge, the stronger are the space charge effects that cause the bunch to spread longitudinally and transversally. In the following, a bunch charge of 0.16 pC, i.e., 10^6^ electrons will be assumed.

### B. Compression of the electron bunch

In order to longitudinally compress the electron bunch, it is necessary to impress a linear velocity chirp onto the bunch, so that particles in the tail move faster than particles in the head of the bunch. This is achieved by passing the beam off-crest, i.e., near the zero crossing of the field, through an RF cavity so that a linear energy chirp is introduced. The compression is limited by the non-linear energy to the velocity relation,^22^ by non-linearities of the RF field^23^ and by the repulsion of the space charge field.

The space charge field also comprises linear and non-linear defocusing components. The non-linear forces depend on the type of distribution and thus can in principle be minimized. An ideal shape for compression is a homogeneously charged ellipsoidal bunch^24^ since the space charge field would be linear in all the directions with such a distribution. However, such a shape is difficult to achieve in practice.

The method we propose will achieve the sub-100 fs time resolution for bunches containing 10^6^ electrons with a non-ellipsoidal and thus less than ideal shape.

## III. SINGLE SHOT UED SETUP

### A. Overview

The proposed design is a half-cell compact RF cavity that would serve two purposes: (1) it would accelerate the electrons to above 130 keV. (2) It would compress the electron bunch to the sub-100 fs duration. This design differs from previous designs in that both the acceleration and the compression can take place inside the same cavity.

Due to the very high gradient present directly at the cathode, a beam with very high particle density could be emitted before reaching the space charge limit. This would lead to a significantly improved transverse beam emittance, however, for uncompressed beams only. In the following, we concentrate thus on a case with reduced charge density at the cathode in favor of a significant longitudinal compression.

The power required for operating the cavity is around 9.2 kW at a temperature of 10 °C, and thus it only requires an RF solid-state amplifier for its operation. In this way, klystrons are avoided as they introduce technical complications in addition to a significant increase in financial expenses.

### B. The RF cavity design

**FIG. 1.**
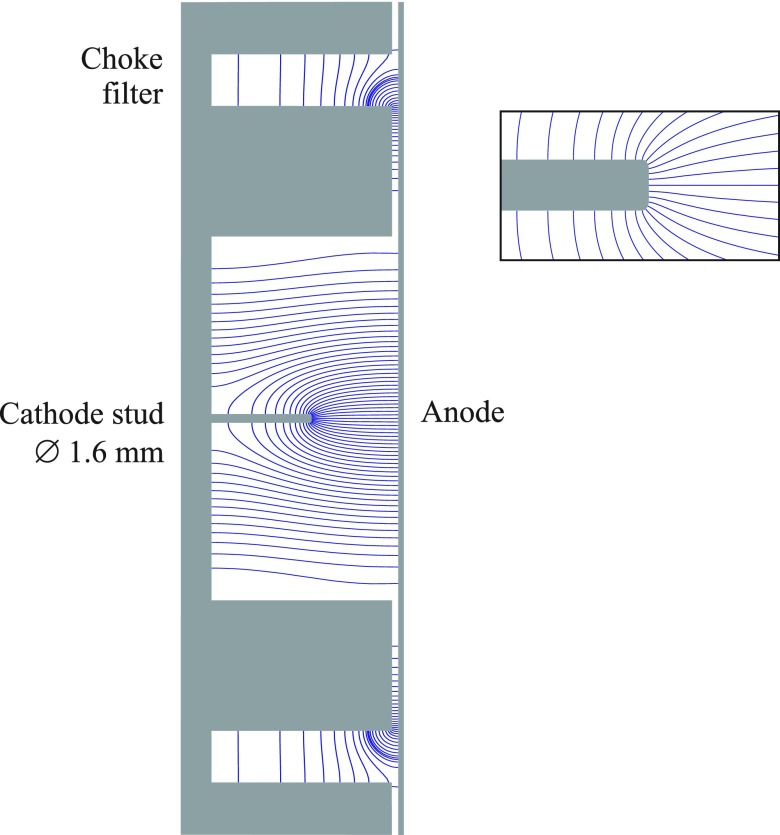
Schematic design of the Compact RF Gun with the cathode stud and the choke filter. The field lines shown in blue are the result of a SUPERFISH calculation. Electrons are released by a laser beam shining onto the cathode and pass through a hole in the anode plane (not shown). The field line density is not proportional to the local field strength. The right panel shows the expanded view of the photocathode region.

**FIG. 2.**
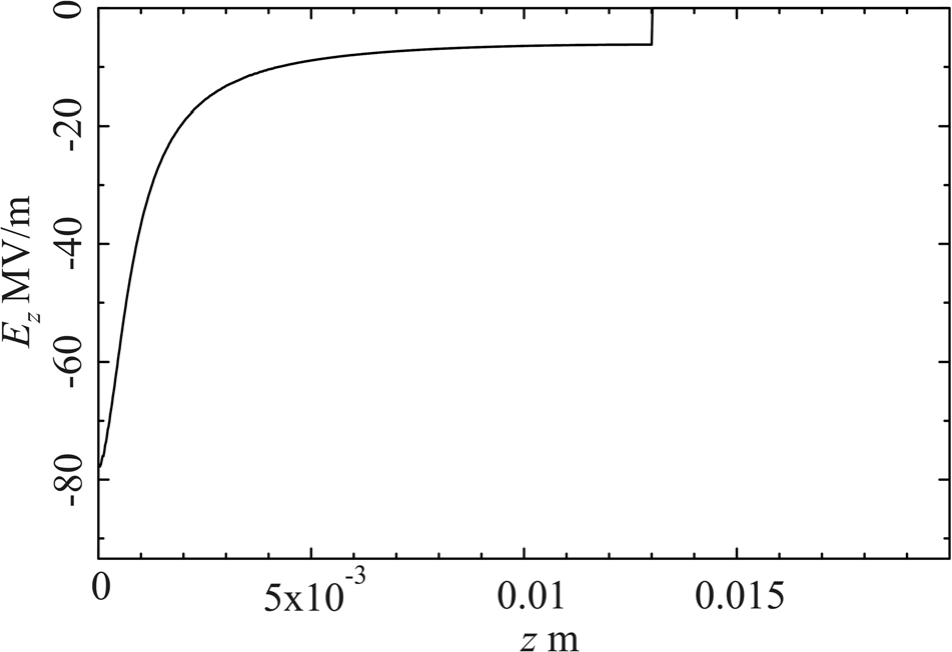
On-axis longitudinal electric field as a function of the longitudinal position *z*. The field is maximal on the cathode surface at *z* = 0.

We have designed the compact RF cavity^32^ with the SUPERFISH code.^25^ A pillbox cavity was considered as the design starting point. It was then modified by introducing a stud into the cavity so as to cause a high field strength of ∼78 MV/m at the cathode which is a flat circular area with a diameter of 1.0 mm at the top of the stud.^26,27^ Subsequently, the cavity was optimized to operate at a resonant frequency of ∼3 GHz and consequently has a diameter of 16 cm. The high field gradient at the cathode serves to both accelerate the electrons and introduce the linear energy profile to achieve maximal compression. As an add-on the cavity contains a ‘choke filter’ which facilitates access to the interior of the cavity as will be discussed in Section III C. Figure 1 shows a cross-section of the RF cavity. Figure 2 shows the on axis longitudinal electric field.

### C. Choke design

The choke filter^28^ is a ring shaped λ-quarter resonator which closes the inner cavity for the 3 GHz design frequency without the necessity of a mechanical contact between the two parts of the cavity. It serves several purposes, one of which is providing easy access to the interior of the cavity as the cavity wall is divided into two pieces: a flat wall where the anode hole is located and the rest of the cavity with a small adjustable gap in between. This helps to equalize the pressure on both the sides of the anode-wall in a vacuum chamber, making a thin wall design possible. Furthermore, it adds more flexibility to the design as it allows for changing the distance between the cathode and the anode. Although not required for single shot UED, the choke filter also serves to damp higher order modes (resonant cavity modes at frequencies higher than the desired 3 GHz frequency).

Moreover, the compact design facilitates operating the cavity at cryogenic temperatures, which would significantly lower the power consumption as well as decrease emittance and improve the beam quality. To produce the same electric field at a temperature of −200 °C, only 3.46 kW is required compared to 9.2 kW at 10 °C.

## IV. PARTICLE TRACKING SIMULATIONS

The electron bunches have been simulated using the ASTRA (A Space charge Tracking Algorithm) code.^29^

### A. Initial conditions

**FIG. 3.**
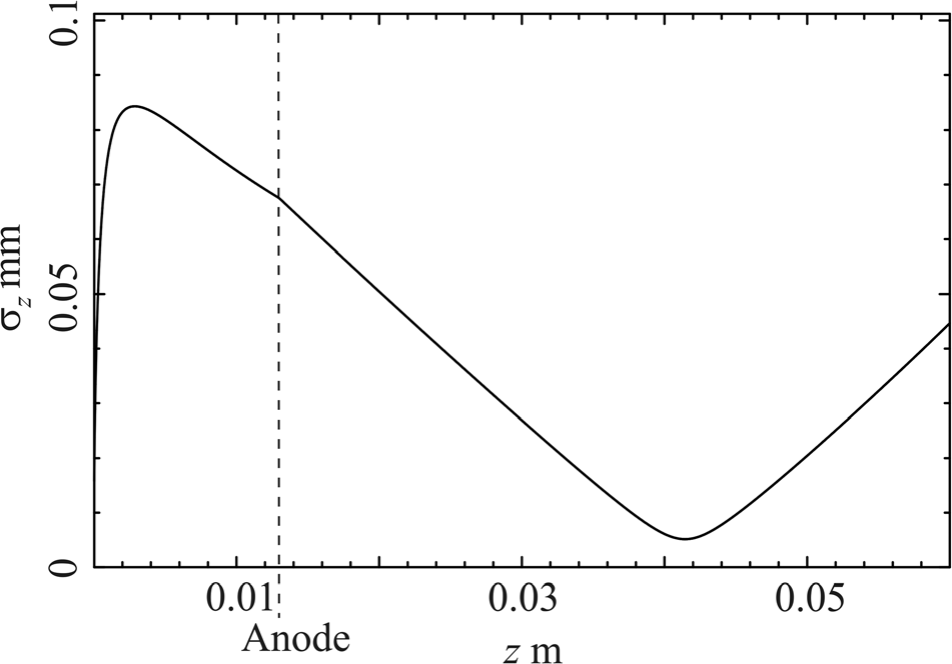
rms bunch length as a function of longitudinal position z.

The electric field achieved in the cavity can accelerate electrons to a maximal energy of 163 keV. However, in order to introduce the energy spread required for the compression of the bunch, the electrons are photo-emitted −36.95° off-crest, so that the mean energy gain is reduced to 137 keV. The initial distribution of the electrons is assumed to form a uniformly filled cylinder with an initial transverse rms size of 60 *μ*m.^30^ This “less than ideal” distribution can be easily achieved by state-of-the-art laser technology. The electrons have a kinetic energy of 0.2 eV and are photo-emitted isotropically at the cathode.^21^ The charge is 0.16 pC. Table I compares the electron pulse length and duration at the sample position for different laser pulse durations. An ideal time resolution at the sample position, about 4 cm downstream of the cathode, is obtained for the 1 ps initial pulse duration. For laser pulses several 100 fs shorter, space charge effects introduce too much non-linear distortions and hence limit the achievable pulse duration at the sample position. For laser pulses several 100 fs longer, the electric RF field curvature is too large to effectively compress the electron pulse. In Figure 3, the bunch length for the 1 ps pulse is plotted as a function of longitudinal position *z*. Mirror charges at the cathode are taken into account.

**TABLE I.** Electron pulse length and duration at the sample position for different initial laser pulse durations.

rms laser pulse duration (fs)	rms electron bunch length (*μ*m)	rms electron pulse duration (fs)
1600	6.28	34.0
1400	5.49	29.7
1200	5.07	27.5
1000	4.99	27.0
800	5.70	30.9
600	7.49	40.6

### B. Transverse dynamics

Transverse focusing is achieved by a solenoid which is located 22 mm in front of the cathode. A second solenoid behind the cathode compensates the field on the cathode. Table II summarizes the achieved transverse spot sizes and coherence lengths at the sample position for different peak solenoid fields up to 0.17 T. Within the parameter range of 0.01 T–0.17 T, the ratio of spot size to coherence length is nearly constant, i.e., the transverse emittance of 47 nm is not affected by the increased charge density in the focused beam condition.

**TABLE II.** Electron beam size and coherence length at the sample position for different values of the solenoid magnetic field strength.

Magnetic field (max. value) (mT)	rms spot size (mm)	Transverse coherence length (nm)
170	0.28	2.0
150	0.35	2.7
130	0.43	3.4
110	0.49	4.0
90	0.55	4.5
70	0.60	4.9
50	0.63	5.2
30	0.66	5.4
10	0.67	5.5

**FIG. 4.**
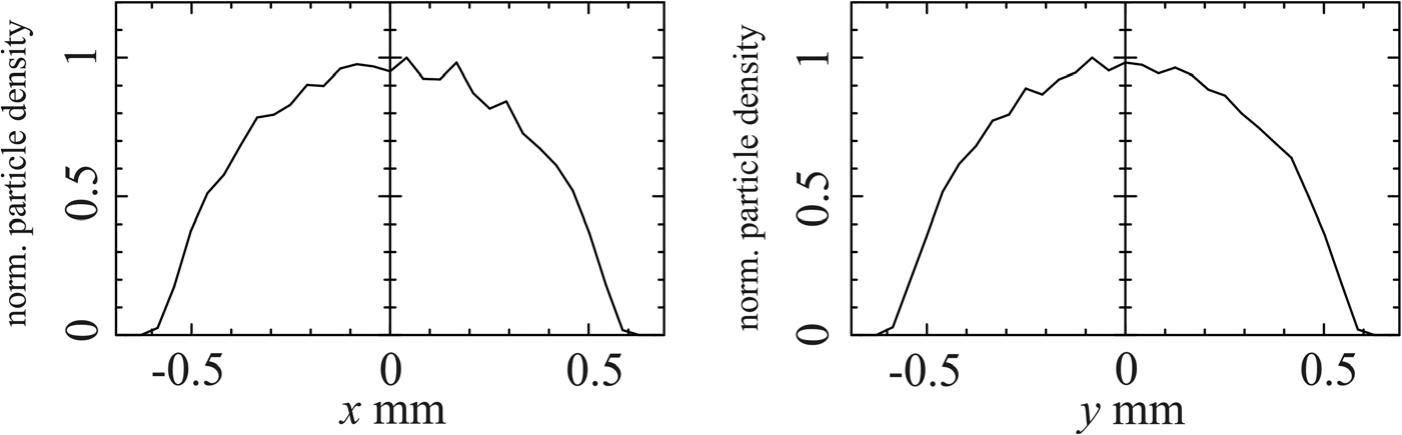
Normalized particle density at the sample position projected onto the transverse x-direction (left) and onto the transverse y-direction (right).

Figure 4 shows the transverse electron distributions at the sample position projected onto orthogonal coordinates for a focusing strength of 0.17 T. The half circle shape shows that the beam does not develop tails and keeps its uniform transverse shape. The maximal electron density in a circle of a diameter of 167 *μ*m is around 9.0 × 10^8^ $\frac{\text{electrons}}{{\text{cm}}^{2}}$. Thus, the beam could be truncated further via an aperture while keeping a high electron density.

The Compact RF Gun is also suitable for other applications where such a high electron density might not be required. In this case, one may lower the solenoid magnetic field strength in favor of a higher coherence length or accelerate more on-crest if the short bunch length is not required. Furthermore, the initial beam size on the cathode can be further reduced, which leads to a reduced emittance.

### C. Time resolution and phase stability considerations

**FIG. 5.**
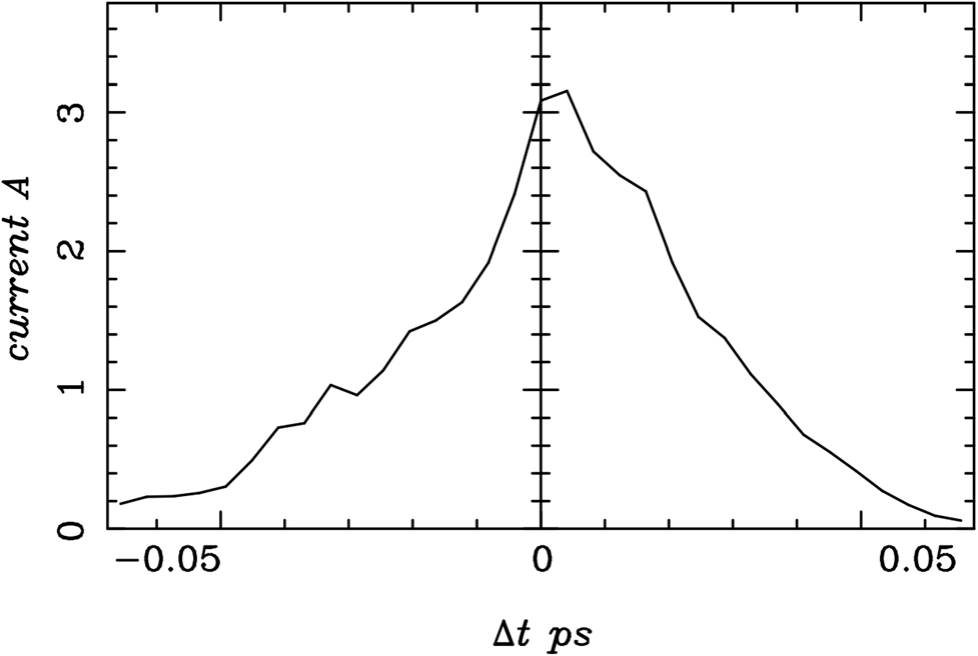
Pulse current at the sample position for the minimal pulse length, cf. Figure 3.

The main source for a timing jitter is the phase instability of the RF. At the injection phase of −36.95°, the time of flight is however only minimally affected by a small change in phase. For a deviation in phase of ±0.04°, the time deviation in the time of flight of the electrons does not exceed 14 fs.^31^ Around 75% of the electrons are contained in a time duration of 58 fs as depicted in Figure 5. This amounts to an effective pulse duration of $\sqrt{58^{2}+14^{2}}\approx 60$ fs or a 60 fs instrument response function. This time resolution is sufficient for virtually all the structural transitions and with sufficient signal to noise ratio, pulse characterization dynamics as short as 10 fs could be extracted from such a source.

## V. CONCLUSION

We have presented a novel design for an RF gun: The Compact RF Gun.

This gun can be used for a variety of applications that require high density electron beams. The coherence length, electron density, and temporal resolution can be adjusted depending on the application. This RF gun is powered by a solid-state amplifier, which is a major advantage as it does not require a klystron and there are no attendant complications in using high power. The required power can be significantly reduced if operated at cryogenic temperatures, which is facilitated by the compact design. It is equally important that this feature will make it possible to specifically exploit cryo-conditions to reduce the transverse emittance. We expect that it will be possible to achieve at least an order of magnitude lower transverse emittance based on recent studies of trialkali photocathodes and temperature dependence. The coherence lengths listed in Table II will be increased significantly or alternatively permit smaller beam focus. In all the cases, the coherence will be sufficient for studying systems as complex as proteins with unit cells on the order of a few nm.

For the specific application of UED, we have shown that the Compact RF Gun concept can produce sub-100 fs electron pulses. High electric field gradients inside the novel half-cell RF cavity are used to simultaneously accelerate and compress electron pulses. With an operation power of 9.2 kW, it can produce 0.16 pC, 137 keV electron pulses with an rms duration of 27 fs (60 fs FWHM), an rms spot size of 276 *μ*m, and a transverse coherence length of 2.0 nm. The relative rms energy spread is <1%. The system is flexible enough to rapidly optimize the photocathode, sample spot size and electron bunch charge and duration as required for specific sample conditions. The electron pulses are suitable for single-shot ultrafast electron diffraction experiments and would open up new time resolution domains in molecular and atomic level structural dynamics research.
